# Higher plasma interleukin − 6 levels are associated with lung cavitation in drug-resistant tuberculosis

**DOI:** 10.1186/s12865-023-00563-2

**Published:** 2023-08-31

**Authors:** Thando Glory Maseko, Slindile Ngubane, Marothi Letsoalo, Santhuri Rambaran, Derseree Archary, Natasha Samsunder, Rubeshan Perumal, Surie Chinappa, Nesri Padayatchi, Kogieleum Naidoo, Aida Sivro

**Affiliations:** 1 Centre for the AIDS Programme of Research in South Africa (CAPRISA), Durban, South Africa; 2 South African Medical Research Council (SAMRC)-CAPRISA-TB-HIV Pathogenesis and Treatment Research Unit, University of KwaZulu-Natal Nelson R Mandela School of Medicine, Durban, South Africa; 3 Department of Medical Microbiology, University of KwaZulu-Natal, Durban, KZN South Africa; 4 JC Wilt Infectious Disease Research Centre, National Microbiology laboratory, Public Health Agency of Canada, Winnipeg, MB Canada; 5 Department of Medical Microbiology and Infectious Diseases, University of Manitoba, Winnipeg, MB Canada

**Keywords:** Tuberculosis, HIV, MDR, XDR, Biomarker, Lung cavitation, Inflammation

## Abstract

**Background:**

Lung cavitation is associated with heightened TB transmission and poor treatment outcomes. This study aimed to determine the relationship between systemic inflammation and lung cavitation in drug-resistant TB patients with and without HIV co-infection.

**Methods:**

Plasma samples were obtained from 128 participants from the CAPRISA 020 Individualized M(X)drug-resistant TB Treatment Strategy Study (InDEX) prior to treatment initiation. Lung cavitation was present in 61 of the 128 drug-resistant TB patients with 93 being co-infected with HIV. The plasma cytokine and chemokine levels were measured using the 27-Plex Human Cytokine immunoassay. Modified Poisson regression models were used to determine the association between plasma cytokine/chemokine expression and lung cavitation in individuals with drug-resistant TB.

**Results:**

Higher Interleukin-6 plasma levels (adjusted risk ratio [aRR] 1.405, 95% confidence interval [CI] 1.079–1.829, p = 0.011) were associated with a higher risk of lung cavitation in the multivariable model adjusting for age, sex, body mass index, HIV status, smoking and previous history of TB. Smoking was associated with an increased risk of lung cavitation (aRR 1.784, 95% CI 1.167–2.729, p = 0.008). An HIV positive status and a higher body mass index, were associated with reduced risk of lung cavitation (aRR 0.537, 95% CI 0.371–0.775, p = 0.001 and aRR 0.927, 95% CI 0.874–0.983, p = 0.012 respectively).

**Conclusion:**

High plasma interleukin-6 levels are associated with an increased risk of cavitary TB highlighting the role of interleukin-6 in the immunopathology of drug-resistant TB.

**Supplementary Information:**

The online version contains supplementary material available at 10.1186/s12865-023-00563-2.

## Background

Lung cavitation is a key pathological feature of *Mycobacterium tuberculosis* (Mtb) infection that results in extensive caseous necrosis. Lung cavities are pathologic gas-filled spaces in lung parenchyma associated with a high bacterial burden resulting in increased transmission, poor drug penetration, and increased morbidity and mortality in people with tuberculosis (TB) [1].

While the mechanism by which cavities are formed is unknown, they are thought to be a consequence of a complex host-pathogen interaction, involving various biochemical, biophysical, microbiological and immunological processes [1]. Data on immunological drivers remain limited, although the inflammatory processes that drive cell necrosis rather than apoptosis during Mtb infection are thought to play a key role in cavity formation [2, 3]. Lung cavitation has several negative consequences for the patient including increased symptom burden, poor drug penetration, longer time to culture conversion, poor treatment outcomes, increased risk of relapse, long-term respiratory impairment, reduced quality of life, and an increased risk for the development of drug resistance [1, 4–6]. Cavitation is shown to promote development of drug resistance in multiple ways, including increasing the frequency of replication-induced mutations [7, 8] through high rates of bacterial proliferation as well as through poor penetration of antimicrobial drugs resulting in pharmacokinetic-pharmacodynamic mismatch [9]. Drug-resistant TB is a major driver of TB related mortality in sub-Saharan Africa, where the incidence of DR-TB is rising. Within sub-Saharan Africa, South Africa has the highest prevalence of multidrug-resistant (MDR)-TB and has recorded the largest outbreak of extensively drug-resistant (XDR)-TB ever reported [10]. Treatment outcomes for DR-TB remain poor, with reports from SA revealing a 70% mortality rate and treatment cure/completion of only 11% after 60 months of follow-up in XDR-TB patients [11]. The presence of lung cavities leads to an increased risk of developing XDR-TB, even among MDR-TB patients on directly observed therapy [12].

The current TB treatment regimens have multiple challenges, including high pill burdens, drug toxicities, prolonged treatment duration and adherence challenges [13]. New approaches to control TB are needed for both diagnosis and treatment, with host-directed therapies being a promising treatment strategy for TB [14]. Identifying blood biomarkers of disease severity and treatment outcome could represent a cost-effective method to personalise treatment and improve treatment outcome. We have previously identified several inflammatory markers associated with TB risk in HIV positive, antiretroviral therapy (ART) experienced individuals (specifically lipopolysaccharide binding protein (LBP), intercellular adhesion molecule (ICAM) -1, interleukin (IL) -1β, IL-6 and IL-1Ra) [15, 16] as well as inflammatory markers associated with disease severity (measured by presence of lung cavitation) in drug-susceptible pulmonary TB (including IL-6 and IL-1Ra) [17]. Here we utilised biological specimens from a well characterised cohort with comprehensive clinical and demographic data to characterise immune markers of lung cavitation in MDR/XDR-TB patients.

## Materials and methods

### Study participants

The study aimed to characterise biomarkers of lung cavitation in MDR/XDR-TB patients and was performed utilising stored plasma specimens from the CAPRISA 020 Individualized M(X)drug-resistant TB Treatment Strategy Study (InDEX). The InDEX study was a randomized controlled clinical trial assessing if a gene-derived individualised treatment approach in patients with drug-resistant TB improved treatment success. Enrolled InDEX study participants were recruited to King Dinuzulu Hospital (KDH) in KwaZulu-Natal, South Africa following a confirmed diagnosis of drug-resistant TB. The presence of lung cavitation was determined by postero-anterior chest radiograph unless a chest radiograph performed 14 days prior to the date of screening was available for review. Study participants were > 18 years with pulmonary TB and Xpert MTB/RIF evidence of rifampicin resistance. For this sub-study, 128 available plasma samples at baseline prior to treatment initiation were evaluated.

### Sample collection and processing

Peripheral blood was collected in acid citrate dextrose (ACD) tubes. Plasma was separated by centrifugation (1600 rpm for 10’) and cryopreserved at − 80 °C until use.

### Cytokine/Chemokine measurement

Cryopreserved plasma samples were thawed and mixed before performing the assays. Cytokine/Chemokine levels were be measured using the Pro Human Cytokine 27-Plex Assay (Bio-Rad, USA) that covers major inflammatory cytokines and analysed on a BioPlex-200 system (Bio-Rad). The Human Cytokine/Chemokine Panel includes the following cytokines and chemokines: fibroblast growth factor (FGF) basic, eotaxin, granulocyte colony-stimulating factor (G-CSF), granulocyte-macrophage colony-stimulating factor (GM-CSF), interferon gamma (IFN-γ), interleukin (IL) -1β, IL-1Ra, IL-2, IL-4, IL-5, IL-6, IL-7, IL-8, IL-9, IL-10, IL-12 (p70), IL-13, IL-15, IL-17 A, interferon γ–Inducible protein-10 (IP-10), monocyte chemoattractant protein-1 (MCP-1), macrophage inflammatory protein (MIP) -1α, MIP-1β, platelet-derived growth factor (PDGF) -BB, regulated on activation normal T cell expressed and secreted (RANTES), *tumor necrosis factor (*TNF) -α, *Vascular endothelial growth factor (*VEGF). All assays were performed following manufacturers’ instructions. Samples with values below the limit of detection (LOD) were assigned the value half of the LOD (LOD/2) in pg/mL.

### Statistical analyses

Statistical analyses were performed using IBM SPSS Statistics version 25, R software version 4.2.2 and the graphs made using GraphPad Prism (V9). All cytokines/chemokines with more than 60% of samples above the limit of detection were log-transformed to reduce variance (IL-1Ra, IL-4, IL-6, IL-7, IL-8, IL-9, IL-13, G-CSF, MCP-1, FGF basic, MIP-1α, MIP-1β, TNF-α, PDGF-BB, Eotaxin, IP-10, RANTES). Cytokines/chemokines with less than 60% detectability were analysed as binary variables (IL-1β, IL-10, IL-12, IL-17, VEGF, GM-CSF, IFN-γ) while cytokines with less than 10% of samples detectable (IL-5, IL-15, IL-2) were excluded from the multivariable analysis to avoid quasi-complete separation. To determine the association between plasma cytokine expression at baseline with disease severity measured by lung cavitation, univariable and multivariable modified Poisson regression models were used. The presence of lung cavitation at baseline was the outcome. Multivariable analyses adjusted for clinical and demographic variables which included age, sex, HIV status, smoking, BMI and history of previous TB. In addition, the use of antiretroviral therapy (ART) was adjusted for when analysing HIV-infected individuals. The association between covariates adjusted for in the multivariable models with lung cavitation was also assessed using modified Poisson regression models.

## Results

### Participant characteristics

The demographic and clinical characteristics of study participants are described in Table 1. The median age was 35 years (IQR 29–41.2), with 57% (73/128) being males. The median body mass index (BMI) was 20.2 kg/m^2^ (IQR 18.5–22.7). Participants with rifampicin-resistant TB and MDR-TB accounted for 89.84% (115/128) of the total cohort while participants with Pre-XDR/XDR-TB accounted for 10.16% (13/128). HIV co-infection was present in 73% (93/128) of study participants with 90.32% (84/93) receiving antiretroviral therapy (ART). The presence of lung cavitation was observed among 47.67% (61/128) of study participants.

**Table 1:** Demographic and clinical characteristics of participants

Variables	Total cohort n = 128	Lung cavitation n = 61	No lung cavitation n = 64
**Age (years), median (IQR)**	35 (29–41.2)	33.0 (29–43)	36 (30–41)
**Female, n (%)**	55 (43)	28 (45.9)	25 (39.1)
**Body mass index (kg/m** ^**2**^ **), median (IQR)**	20.2 (18.5–22.7)	19.8 (17.9–21.5)	20.5 (19.0–23.6)
**HIV Positive, n (%)**	93 (72.7)	37 (60.7)	53 (82.8)
**ARV status**^**a**^, **n (%)**			
No	9 (7)	4 (6.6)	4 (6.2)
Yes	84 (65.6)	33 (54.1)	49 (76.6)
**Previous TB History, n (%)**			
No	77 (60.2)	37 (60.7)	38 (59.4)
Yes	51 (39.8)	24 (39.3)	26 (40.6)
**TB diagnosis**			
RR/MDR	115 (89.8)	57 (93.4)	56 (87.5)
Pre-XDR/XDR	13 (10.2)	4 (6.6)	8 (12.5)
**Alcohol use, n (%)**			
No	118 (92.2)	53 (86.9)	62 (96.9)
Yes	10 (7.8)	8 (13.1)	2 (3.1)
**Smoking, n (%)**			
No	120 (93.8)	57 (93.4)	60 (93.8)
Yes	8 (6.2)	4 (6.6)	4 (6.2)
Lung cavities*, n (%)			
None	64 (50)	-	64 (100)
Unilateral	46 (35.9)	46 (75.41)	-
Bilateral	15 (11.7)	15 (24.59)	-

^#^ Values for clinical and demographic variables reported at baseline

*Three participants with missing lung cavitation status

^a^ 34 participants with missing ARV status

### Effect of plasma cytokine/chemokine expression on presence of lung cavitation

Modified Poisson regression models were used to assess the association between plasma cytokine/chemokine expression and lung cavitation in individuals with DR-TB. Following univariable and multivariable analyses adjusting for age, sex, BMI, HIV status, smoking and previous history of TB; increased plasma expression of IL-6 [aRR 1.405, 95% CI 1.079–1.829, p = 0.011] was associated with an increased risk of lung cavitation in the total cohort. A unit increase in the log expression of IL-6 led to a 41% increase in the risk of lung cavitation (Table 2). Similarly, a trend was observed in the sub-analysis of HIV positive participants controlling for ART use [aRR 1.424, 95% CI 0.991–2.046, p = 0.056 (Additional File 1).

Multivariable models that fitted lung cavitation against the adjusted variables (age, sex, BMI, HIV status, smoking and previous history of TB) in the total cohort, showed that HIV positive participants had a significant 46% reduction in the risk of lung cavitation in comparison to HIV negative participants [aRR 0.536, 95% CI 0.371–0.775, p = 0.001]. The risk of lung cavitation was 7% lower for each unit increase in BMI [aRR 0.927, 95% CI 9.874–0.983, p = 0.012] while males had a 44% lower risk of lung cavitation [aRR 0.559, 95% CI 0.381–0.821, p = 0.003]. Smoking was associated with an increased risk of lung cavitation [aRR 1.784, 95% CI 1.167–2.729, p = 0.008] (Additional File 1).

**Table 2:** Association between plasma cytokine/chemokine expression with lung cavitation among HIV positive and negative participants with DR-TB (n = 125)

Cytokine/ Chemokine	Univariable				Multivariable		
	**RR**	**95% CI**	**p-value**		**aRR**	**95% CI**	**p-value**
IL-1Ra	1.158	0.761–1.760	0.494		1.115	0.736–1.689	0.607
IL-4	0.941	0.620–1.426	0.773		1.127	0.742–1.713	0.575
IL-6	1.347	1.006–1.803	**0.045**		1.405	1.079–1.829	**0.011**
IL-7	0.871	0.713–1.064	0.176		0.922	0.756–1.125	0.425
IL-8	1.019	0.557–1.863	0.951		0.967	0.583–1.603	0.896
IL-9	1.025	0.535–1.964	0.940		1.112	0.601–2.059	0.735
IL-13	0.853	0.628–1.157	0.307		0.851	0.624–1.161	0.309
G-CSF	0.967	0.821–1.139	0.689		0.987	0.850–1.147	0.868
MCP-1	0.951	0.749–1.208	0.682		1.072	0.828–1.387	0.597
FGF	1.393	0.659–2.944	0.386		1.324	0.614–2.859	0.474
MIP-1α	1.183	0.959–1.458	0.116		1.139	0.924–1.403	0.222
MIP-1β	1.084	0.530–2.218	0.825		1.199	0.609–2.361	0.600
TNF-α	0.934	0.635–1.375	0.731		1.097	0.766–1.572	0.614
PGDF-BB	1.075	0.802–1.441	0.628		1.175	0.891–1.550	0.253
Eotaxin	1.044	0.688–1.586	0.839		1.206	0.795–1.832	0.378
IP-10	1.039	0.797–1.354	0.778		1.116	0.880–1.414	0.366
GM-CSF	0.926	0.574–1.492	0.752		0.902	0.591–1.376	0.631
IFN-γ	0.751	0.502–1.124	0.164		0.808	0.552–1.185	0.275
IL-10	1.177	0.796–1.740	0.414		1.131	0.761–1.681	0.544
IL-12	0.868	0.607–1.241	0.438		0.887	0.627–1.256	0.499
IL-17	1.115	0.779–1.596	0.553		1.108	0.788–1.557	0.556
IL-1b	1.045	0.730–1.498	0.809		0.958	0.678–1.354	0.809
VEGF	1.155	0.741–1.801	0.525		1.176	0.792–1.744	0.378
RANTES	0.767	0.542–1.085	0.134		0.853	0.608–1.198	0.359

*Three participants were excluded due to missing lung cavitation status

## Discussion

Cavitary TB is a serious consequence of pulmonary TB, and is associated with increased morbidity and mortality, the development of drug resistance and higher transmission rates [1]. In this study, we assessed the association between plasma cytokine/chemokine expression and lung cavitation among individuals with DR-TB. Increased plasma expression of IL-6 was associated with higher risk of lung cavitation among individuals with DR-TB. HIV positive participants, males and participants with higher BMI had a reduced risk of lung cavitation in participants with DR-TB. Additionally, Individuals who smoke had an increased risk of lung cavitation.

IL-6 is a pleiotropic cytokine with dual pro- and anti-inflammatory properties. IL-6 activates target cells either via membrane–bound IL-6R (classic signalling) or through soluble forms of IL-6R (sIL-6R, trans-signalling), the latter of which was shown to account for its proinflammatory properties [18]. IL-6 modulates innate and adaptive immunity [19]. It is secreted by various immune cells including macrophages, neutrophils and lung epithelial cells at sites of inflammation. IL-6, is induced by Mtb infection, [20] and has both positive and negative effects on host immune control of Mtb [21]. We have previously shown that plasma IL-6 is associated with both shorter time to culture conversion as well as increased risk of TB recurrence and presence of lung cavitation in patients with drug susceptible TB, highlighting its dual role in TB [15, 16]. Increased IL-6 levels measured in bronchoalveolar lavage (BAL) of patients with pulmonary TB were shown to correlate with lung cavitation [22]. Additionally, BAL IL-6 expression has previously been correlated with a chest high-resolution computed tomography score which factored cavities, miliary nodules and bronchial wall thickening among HIV negative patients with active TB [22]. IL-6 has also been implicated in promoting fibroblast development and subsequently, pulmonary fibrosis [23, 24]. Elevated IL-6 expression was also reported in pleural fluid of TB patients as well as in cerebrospinal fluid (CSF) in patients with TB meningitis [25–27]. While IL-6 is predominantly associated with disease severity in TB, knockout studies have shown that IL-6 plays an important role in controlling bacterial load early in the infection through induction of protective interferon gamma-producing T cell responses [28].

The COVID-19 pandemic has highlighted the use of host directed therapies targeting IL-6. This includes biological agents that target the IL-6 itself (clazakizumab, olokizumab, sirukumab and siltuximab), its receptor (tocilizumab and sarilumab) and drugs that inhibit IL-6 *trans*-signalling by blocking the soluble IL-6 receptor (sIL-6R) (olamkicept) [29, 30]. These IL-6 inhibitors vary in potency, have distinct pharmacodynamics and result in distinct downstream effects. While cytokine and receptor blockers are known to inhibit the classic IL-6 pathway and result in significant immunosuppression, the sIL-6R inhibitor, olamkicept was shown to exclusively block IL-6 proinflammatory trans-signalling without affecting IL-6 classic signalling and causing immunosuppression [30]. As seen with the use of IL-6 targeting in COVID-19, the efficacy of any IL-6 therapy would depend on the severity of the disease and the timing of the intervention; as well as the ability to distinguish between IL-6 as a cause or a consequence of the disease.

Smoking was found to increase the risk of lung cavitation among DR-TB patients. Smoking is known to be associated with more extensive lung disease and cavitation as well as poor treatment outcome in pulmonary TB [31, 32]. HIV status, increased BMI and male sex were associated with reduced risk of lung cavitation. Previous studies have reported lower odds of lung cavitation in HIV positive individuals with pulmonary TB in comparison to HIV negative individuals and this was related to immunosuppression and the reduction in CD4^+^ T cell counts that are thought to play a key role in lung immunopathology during Mtb infection [33–36]. In addition to lower prevalence of cavitary disease HIV positive individuals are more likely to have acid-fast smear negative disease, as well as to have extrapulmonary TB, all of which are associated with delays in TB diagnosis and poor treatment outcomes [37–39]. Higher odds of lung cavitation were reported among TB cases with severe malnutrition (BMI < than 16 kg/m^2^) in comparison to individuals with a BMI greater than or equal than 18.5 kg/m^2^ [40]. Weight loss is a clinical manifestation of TB and is known to increase following TB treatment [41] however, a lack of adherence to MDR-TB treatment leads to slower increases in BMI [42]. Contrasting results have been reported on the relationship between gender and TB disease severity. Greater lung involvement was reported in males in comparison to females with pulmonary TB using Ralph scoring [43] with pulmonary fibrosis also being more frequent among males in comparison to females [44]. The relationship between gender and lung cavitation among drug-resistant TB patients remains to be further characterised.

Previous studies have highlighted the role of IL-6 in drug-susceptible TB, however studies on cytokine signatures and their role in DR-TB are limited. Here we highlight the association between plasma IL-6 levels and presence of lung cavitation in a well-characterised cohort of individuals with active DR-TB. This study has several limitations, including a lack of access to viral load and CD4 counts for HIV positive individuals with DR-TB at sampling timepoint. Additionally, we were not able to determine whether the lung cavitation was related to previous TB disease or the current episode of TB. While our results show an association between plasma IL-6 and presence of cavitary disease in DR-TB, we are unable to distinguish between IL-6 as a consequence or a cause of the disease. Further studies are needed to determine the relationship between lung cavitation and IL-6 signalling and the potential of IL-6 blocking agents in TB treatment.

### Electronic supplementary material

Below is the link to the electronic supplementary material.

Additional File 1: Supplementary Table 1 and Supplementary Figure 1

## Data Availability

The datasets used and/or analysed during the current study are available from the corresponding author on reasonable request.

## Abbreviations

ACD: Acid citrate dextrose
aRR: Adjusted risk ratio
ART: Antiretroviral therapy
BAL: Bronchoalveolar lavage
BMI: Body mass index
CAPRISA: Centre for the AIDS Programme of Research in South Africa
CI: Confidence interval
CSF: Cerebrospinal fluid
DR-TB: Drug resistant tuberculosis
FGF: Fibroblast growth factor
G-CSF: Granulocyte colony-stimulating factor
GM-CSF: Granulocyte-macrophage colony-stimulating factor
HIV: Human immunodeficiency virus
ICAM: Intercellular adhesion molecule-1
IFN-γ: Interferon gamma
IL: Interleukin
InDEX: The Individualized M(X) drug-resistant TB treatment Strategy Study
IP-10: Interferon gamma-induced protein 10
IQR: Interquartile range
KDH: King Dinuzulu hospital
LBP: lypopolysaccharide binding protein
LOD: Limit od detection
MCP-1: Monocyte chemoattractant protein-1
MDR: Multidrug-resistant
MIP: Macrophage inflammatory protein
Mtb: Mycobacterium tuberculosis
PDGF-BB: Platelet-derived growth factor-BB
RANTES: Regulated on activation, normal T cell expressed and secreted
RR: Risk ratio
TB: Tuberculosis
TNF: Tumor necrosis factor
VEGF: Vascular endothelial growth factor
XDR: Extensively drug-resistant TB
