# Supplementary material for: Higher plasma interleukin − 6 levels are associated with lung cavitation in drug-resistant tuberculosis
Source: BMC Immunol. 2023 Aug 31;24:26. doi: 10.1186/s12865-023-00563-2 (PMC10472663; doi:10.1186/s12865-023-00563-2)
Supplement: Supplementary file 1 — Additional File 1: Supplementary Table 1 and Supplementary Figure 1 [file 12865_2023_563_MOESM1_ESM.docx]

**Supplemental materials**

**Supplementary Table 1**. Association between plasma cytokine/chemokine expression with lung cavitation among HIV positive participants with DR-TB

| **Cytokine/** | **Univariable** | | |  | **Multivariable** | | |
| --- | --- | --- | --- | --- | --- | --- | --- |
| **Chemokine** | **RR** | **95% CI** | **p-value** |  | **aRR** | **95% CI** | **p-value** |
| IL-1Ra | 1.180 | 0.693 – 2.009 | 0.541 |  | 1.098 | 0.653 – 1.848 | 0.724 |
| IL-4 | 0.904 | 0.498 – 1.643 | 0.742 |  | 0.968 | 0.533 – 1.757 | 0.914 |
| IL-6 | 1.471 | 0.997 – 2.171 | 0.052 |  | 1.424 | 0.991 – 2.046 | 0.056 |
| IL-7 | 0.814 | 0.619 – 1.071 | 0.142 |  | 0.840 | 0.639 – 1.103 | 0.210 |
| IL-8 | 0.805 | 0.328 – 1.977 | 0.637 |  | 0.704 | 0.305 – 1.622 | 0.410 |
| IL-9 | 1.255 | 0.506 – 3.112 | 0.624 |  | 1.413 | 0.556 – 3.591 | 0.467 |
| IL-13 | 0.952 | 0.621 – 1.460 | 0.823 |  | 0.926 | 0.590 – 1.453 | 0.739 |
| G-CSF | 0.903 | 0.713 – 1.144 | 0.398 |  | 0.877 | 0.706 – 1.090 | 0.238 |
| MCP1 | 1.008 | 0.721 – 1.411 | 0.961 |  | 1.112 | 0.792 – 1.561 | 0.541 |
| FGF | 1.802 | 0.549 – 5.914 | 0.332 |  | 1.783 | 0.500 – 6.359 | 0.373 |
| MIP-1α | 1.158 | 0.844 – 1.588 | 0.363 |  | 1.080 | 0.792 – 1.474 | 0.626 |
| MIP-1β | 1.397 | 0.515 – 3.791 | 0.512 |  | 1.561 | 0.557 – 4.375 | 0.397 |
| TNF-α | 0.836 | 0.461 – 1.515 | 0.555 |  | 0.896 | 0.531 – 1.510 | 0.679 |
| PGDF-BB | 1.022 | 0.652 – 1.603 | 0.924 |  | 1.157 | 0.768 – 1.743 | 0.486 |
| Eotaxin | 1.201 | 0.699 – 2.061 | 0.507 |  | 1.339 | 0.771 – 2.324 | 0.300 |
| IP-10 | 1.144 | 0.820 – 1.594 | 0.429 |  | 1.149 | 0.848 – 1.557 | 0.369 |
| GM-CSF | 0.872 | 0.456 – 1.668 | 0.679 |  | 0.814 | 0.448 – 1.479 | 0.500 |
| IFN-γ | 0.677 | 0.398 – 1.152 | 0.151 |  | 0.687 | 0.407 – 1.160 | 0.160 |
| IL-10 | 1.145 | 0.665 – 1.971 | 0.626 |  | 1.073 | 0.627 – 1.834 | 0.797 |
| IL-12 | 0.906 | 0.547 – 1.502 | 0.703 |  | 0.869 | 0.533 – 1.415 | 0.571 |
| IL-17 | 1.062 | 0.648 – 1.743 | 0.810 |  | 1.062 | 0.646 – 1.747 | 0.813 |
| IL-1b | 0.892 | 0.537 – 1.480 | 0.657 |  | 0.791 | 0.474 – 1.320 | 0.370 |
| VEGF | 1.185 | 0.665 – 2.111 | 0.566 |  | 1.126 | 0.675 – 1.880 | 0.649 |
| RANTES | 0.826 | 0.505 – 1.350 | 0.445 |  | 0.897 | 0.557 – 1.444 | 0.654 |

*Three participants were excluded due to missing lung cavitation status

Relative risk

0.5

1.0

1.5

2.0

2.5

3.0

HIV infected

Smoker

Previous history of TB

BMI (kg/m^2^)

Age (years)

Males


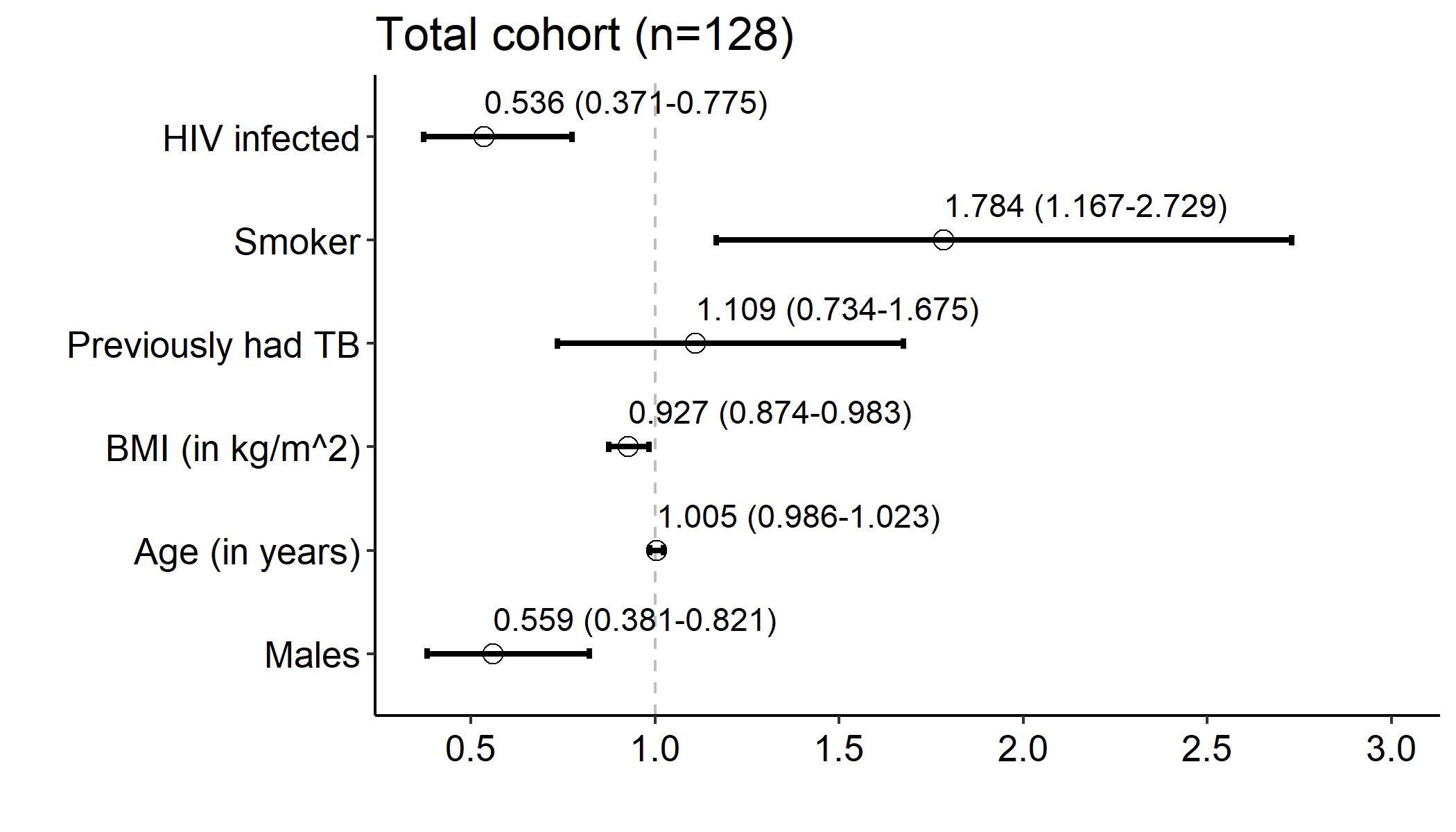


0.536 (0.371 – 0.775)

1.784 (1.167 – 2.729)

1.109 (0.734 – 1.675)

.927 (0.874 – 0.983)

1.005 (0.986 – 1.023)

0.559 (0.381 – 0.821)

0

**Supplementary Figure 1**. Relative risk for covariates in a multivariable Poisson model prior correcting for cytokines/chemokines (n=125)
